# Hybrid Nonviral Nanocarriers Enable Functional Neural Modulation

**DOI:** 10.1021/acsnano.6c07588

**Published:** 2026-07-22

**Authors:** Eunji Hong, Xinxin Xu, Sizhe Huang, Geunho Jang, Zuer Wu, Qianbin Wang, Siyuan Rao

**Affiliations:** † Department of Biomedical Engineering, 14787 Binghamton University, State University of New York, Binghamton, New York 13902, United States; ‡ Integrative Neuroscience Program, Binghamton University, State University of New York, Binghamton, New York 13902, United States

**Keywords:** nonviral nanocarriers, exosomes, liposomes, gene delivery, neural modulation, optogenetics

## Abstract

Extracellular vesicles released from the neuronal cells mediate the transfer of proteins, nucleic acids, and neurotransmitter-related cargoes, shaping gene expression and intercellular communication across neural circuits. Leveraging this endogenous pathway, we fused astrocyte-derived exosomes with RNA-loaded synthetic liposomes to create sub-100 nm hybrid nanoparticles for central nervous system delivery. This design addresses key limitations of existing systems: conventional liposomes lack cell-type specificity and can be toxic, whereas native vesicles are difficult to load efficiently. By tuning a mildly cationic surface, the hybrids support efficient gene transfer to neurons without disrupting membrane integrity or inducing measurable cytotoxicity. We validated this platform in vivo by delivering Cre recombinase mRNA in transgenic mice and inducing channel rhodopsin-2 expression in the motor cortex and ventral tegmental area, yielding behavioral changes during optogenetic stimulation.

## Graphical abstract

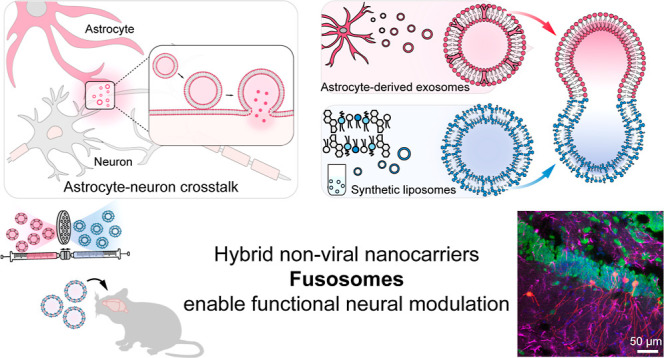

## Introduction

Cellular communication in the nervous system is mediated not only by synapses and paracrine signals but also by extracellular vesicles (EVs), membrane-bound nanoparticles that transport proteins, nucleic acids, lipids, and metabolites to shape neuronal and glial function.^1–4^ Their parent-cell-derived membranes carry tetraspanins, adhesion molecules, and fusogenic proteins that enable selective targeting and efficient cargo delivery to certain cell types, making EVs attractive candidates for nonviral gene delivery.^2,5,6^ However, native EVs carry heterogeneous and poorly controlled cargo, and conventional loading methods often compromise encapsulation efficiency and vesicle integrity.^4,7–9^

Synthetic liposomes offer precise, scalable control over composition and payload but lack intrinsic biological identity, leading to off-target distribution and toxicity, particularly in the brain.^10–12^ Recent advances have further improved central nervous system (CNS) delivery through peptide functionalization,^13^ blood–brain barrier (BBB)-crossing ionizable lipids,^14^ and optimized systemic mRNA delivery formulations.^15^ These studies highlight the rapid progress of synthetic LNPs for brain delivery. However, synthetic systems still generally rely on extensive formulation screening to balance delivery efficiency, tissue tropism, off-target distribution, and biocompatibility. A hybrid EV-liposome strategy may therefore provide a complementary route by combining synthetic cargo-loading control with exosome-derived membrane features that support biological interactions with neural cells.

To integrate biological specificity with synthetic precision, we developed Fusosomes, hybrid nanoparticles formed by fusing astrocyte-derived exosomes with gene-loaded liposomes. Fusosomes preserve the native exosomal membrane and its targeting and fusion machinery while using the synthetic compartment to define payload and physicochemical properties.^15–19^ By tuning lipid composition, surface charge, and size, Fusosomes can be engineered as sub-100 nm carriers that balance EV-like trafficking with the controllability of synthetic nanocarriers.^20–23^

## Results/Discussion

Fusosomes were designed and prepared by physically fusing mouse astrocyte-derived exosomes with synthetic liposomes preloaded with defined genetic cargo (Figure 1). Exosomes from cultured mouse astrocytes were first harvested by ultracentrifugation and characterized by transmission electron microscopy (TEM) and dynamic light scattering (DLS) analysis (Figure 2A,B). mRNA-loaded synthetic liposomes were prepared using thin-film hydration followed by ultrasonication. By adapting the method from prior studies,^24,25^ sequential ultrasonication of exosome-liposome mixtures and membrane extrusion produced Fusosome particles. Native exosomes (Exo) displayed an average diameter of 64.16 ± 7.48 nm, whereas synthetic liposomes (Lipo) measured 98.35 ± 9.35 nm. After fusion, Fusosomes (Fuso) exhibited uniform, spherical morphologies with a mean diameter of 95.16 ± 1.69 nm. This fusion strategy can be readily extended to tune surface charge by incorporating charged lipid molecules or surface polymers. Drawing on commonly used transfection agents, we introduced a cationic surface on synthetic liposomes using linear polyethylenimine (PEI; MW 25,000)^26–28^ during liposome preparation to generate PEI-modified liposomes (PEI/Lipo), which were subsequently fused with exosomes to produce PEI/Fusosomes (PEI/Fuso) with a similar diameter of 109.0 ± 4.17 nm (Figure 2C). When Exo with a zeta potential of 17.10 ± 0.52 mV were fused with Lipo with a zeta potential of 2.66 ± 0.14 mV, the resulting Fuso exhibited a moderately negative surface charge of −5.64 ± 0.35 mV. After PEI modification of the lipid layer, the zeta potential of PEI/Fuso shifted to near neutral at −0.07 ± 0.34 mV (Figure 2D). Fuso formulations remained stable for at least 2 weeks at 4 °C, with changes in hydrodynamic diameter of less than 40 nm over this period (Figure 2E). The size distribution, zeta potential shift, TEM morphology, and colloidal stability support Fusosome formation after ultrasonication and extrusion. However, these population-level measurements do not directly quantify the yield of fully fused hybrid particles versus residual unfused exosomes or liposomes. Future studies using FRET-based lipid mixing, dual-label single-particle analysis, or lipid/protein compositional profiling could be useful to directly measure fusion efficiency and membrane uniformity.^25,29,30^

**Figure 1:**
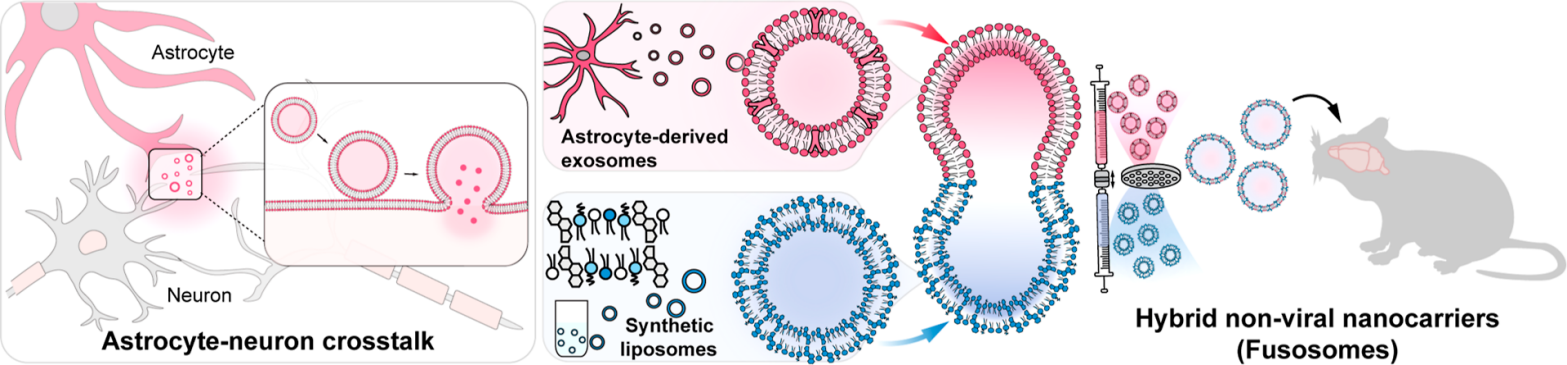
Engineering hybrid nanovesicles for enhanced mRNA delivery through the fusion of astrocyte-derived exosomes and synthetic liposomes. Schematic illustration of hybrid nonviral nanocarriers (Fusosomes) engineered through the fusion of astrocyte-derived exosomes with biomimetic vesicles, liposomes, designed to enhance mRNA delivery. The hybrid system integrates the intrinsic neuron-targeting properties of astrocyte-derived exosomes with the tailored composition of synthetic liposomes, facilitating efficient delivery of Cre recombinase mRNA for optogenetic manipulation.

**Figure 2:**
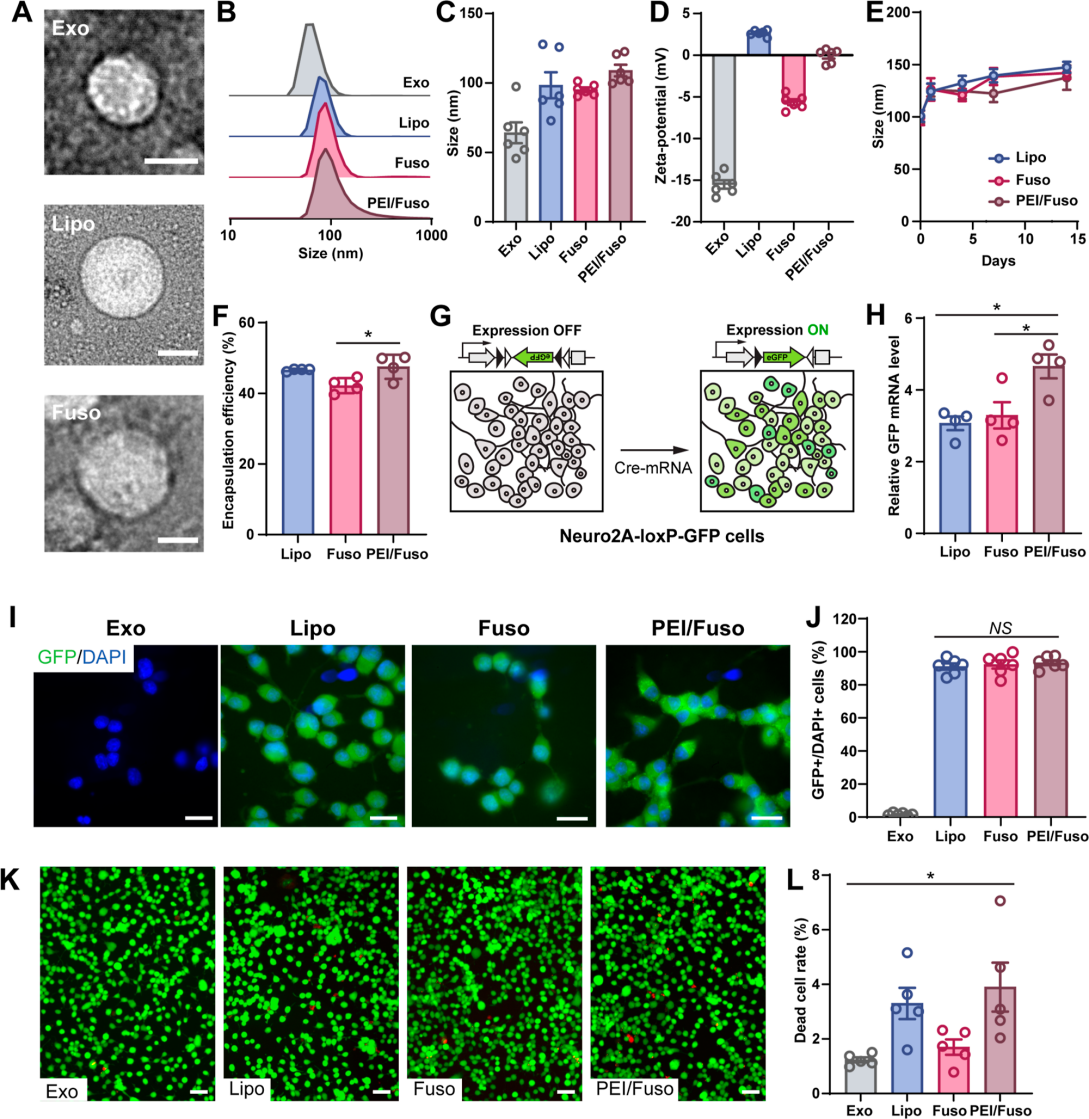
Physicochemical characterization and in vitro functional analysis of Fusosomes for mRNA delivery. (A) Transmission electron microscopy (TEM) images of liposomes (Lipo), astrocyte-derived exosomes (Exo), and hybrid nanovesicles (Fusosome; Fuso). Scale bar: 50 nm. (B–D) Physicochemical characterization of hybrid nanovesicles using dynamic light scattering (DLS) (Exo; gray, Lipo; blue, Fuso; pink, PEI-modified Fusosome (PEI/Fuso); dark magenta, *n* = 6 individual samples in saline): size distribution profiles (B), comparative hydrodynamic diameter distributions showing relative particle number (%) (C), and zeta potential measurements of each formulation (D). (E) Storage stability assessment of particle size distribution over 2 weeks at 4 °C (*n* = 4 individual samples). (F) mRNA encapsulation efficiency (%) comparing the loading capacity of Cre recombinase encoding mRNA across different formulations (*n* = 4 individual samples). (G) Schematic illustration of the Cre/loxP gene expression system in Neuro2A-loxP-GFP cells, regulated by Cre mRNA, which encodes Cre recombinase. (H) Quantification of GFP mRNA expression by RT-qPCR, normalized to untreated control (*n* = 4 independent biological replicates). (I) Representative fluorescence microscopy images showing GFP expression in Neuro2A-loxP-GFP cells after 48 h transfection with each formulation, with DAPI nuclear staining. Scale bars: 20 μm. (J) Quantification of GFP-positive cells normalized to DAPI-positive nuclei. (*n* = 6 individual biological replicates). (K) Representative fluorescence microscopy images from live/dead cell assays to evaluate biocompatibility (live: green; dead: red). Scale bar: 50 μm. (L) Quantitative analysis of cell death rates determined from five independent images. Data presented as mean ± SEM. Statistical significance was determined using one-way ANOVA with Tukey’s multiple comparisons test (F, Fuso vs PEI/Fuso *p = 0.0250, H, Lipo vs PEI/Fuso *p = 0.0129; Fuso vs PEI/Fuso *p = 0.0286, J, NS *p* = 0.7256, L, Exo vs PEI/Fuso *p = 0.0170).

We next assessed mRNA encapsulation efficiency and delivery efficacy across formulations. Through RiboGreen RNA assay,^19,31^ we found that Fuso exhibited an encapsulation efficiency of 42.17 ± 1.06%, a slight decrease from 46.62 ± 0.17% for Lipo, whereas cationic surface modification with PEI modestly enhanced mRNA loading to 47.51 ± 1.71% (Figure 2F). To evaluate functional delivery, we incubated Cre mRNA-encapsulating nanocarriers with engineered Neuro2A-loxP-GFP cells,^32,33^ in which Cre recombinase activates GFP transcription (Figure 2G). Neuro2A cells treated with astrocyte-derived exosomes without Cre mRNA, Lipo-Cre, Fuso-Cre, or PEI/Fuso-Cre were harvested and analyzed for GFP mRNA expression by quantitative PCR. Relative to the minimal eGFP mRNA level observed in exosome-treated cells (1.09 ± 0.19), the Lipo and Fuso groups showed approximately 3-fold higher expression, 3.23 ± 0.22 and 3.12 ± 0.33, respectively, whereas the PEI/Fuso group reached more than 4-fold higher levels (4.33 ± 0.41) (Figures 2H and S1). Fluorescence microscopy confirmed robust GFP expression in Neuro2A cells 24 h after transfection, and GFP expression remained stable over time (Figure 2I). Time-dependent fluorescence imaging at 12, 24, and 48 h further demonstrated the temporal progression and sustained GFP expression following Cre mRNA delivery in vitro (Figure S2). Fluorescent microscopy quantification of GFP-positive cells showed a minimal percentage in Exo-treated cells (1.51 ± 0.33%), compared with more than 90% in all three lipid-based groups: 91.10 ± 1.91% (Lipo), 92.33 ± 2.51% (Fuso), and 93.41 ± 1.51% (PEI/Fuso) (Figure 2J). Although PEI/Fuso yielded the highest GFP mRNA levels, GFP expression at the single-cell level was similar across all lipid-based formulations. This observation suggests that surface charge and membrane fusion play key roles in governing intracellular delivery and gene delivery. To evaluate biocompatibility with neuronal cells, we conducted live/dead cell viability using Calcein AM/ethidium homodimer-1 assay^34^ at high lipid doses (300 μg mL^–1^). Exo-, Lipo-, and Fuso-treated cells showed minimal cytotoxicity with 1.23 ± 0.09%, 3.3 ± 0.57% and 1.7 ± 0.28% dead cells, respectively, whereas PEI/Fuso caused 3.95 ± 0.90% cell death (Figure 2K,L). Although PEI/Fuso improves mRNA loading and transcriptional output, its modest increase in cell death indicates an efficiency-biocompatibility trade-off. For chronic neural modulation, this trade-off requires careful consideration because long-term applications prioritize neuronal viability, preserved membrane properties, and minimal cumulative toxicity in addition to delivery efficiency.^35–37^ Thus, PEI/Fuso may be useful for short-term applications requiring higher transient expression or dose-sparing delivery, whereas unmodified Fuso provides a more balanced profile for repeated or longer-term neural studies.

Since the fusion strategy primarily relies on nanocarrier membrane manipulation,^38,39^ we next tested whether Fusosome-based gene delivery alters the membrane properties of recipient cells.^40,41^ We characterized the membrane properties of Neuro2A-loxP-GFP cells following Cre-mRNA (0.5 μg/mL) delivery via nanocarrier incubation over 48 h (Figure 3A). Whole-cell patch-clamp recordings showed that untreated Neuro2A cells displayed a baseline resistance of 775.6 ± 47.03 MΩ, whereas Exo-, Lipo-, and Fuso-treated groups exhibited modestly increased resistances of 921.0 ± 75.78 MΩ, 890.8 ± 54.93 MΩ, and 1108.0 ± 137.2 MΩ, respectively. After introducing the cationic polymer composite, the PEI/Fuso-treated group showed a further increase in membrane resistance to 1512 ± 189.0 MΩ (Figure 3B). To evaluate the ability of the membrane to change potential in response to current, which affects action potential dynamics, we also measured membrane capacitance. No significant differences in whole-cell capacitance were observed across all nanocarrier-treated groups (Figure 3C), nor in the normalized membrane capacitance relative to cell surface area (Figure 3D). Resting membrane potential is a key parameter for assessing cell health and, in neurons, is closely linked to excitability.^40,42^ We therefore characterized the resting membrane potential in current-clamp mode. Untreated Neuro2A cells had a baseline potential of −7.85 ± 1.42 mV, whereas nanocarrier-treated groups exhibited more hyperpolarized membrane potentials. The Exo-treated group shifted to −11.34 ± 1.58 mV, the Fuso-treated group to −12.40 ± 2.80 mV, and the PEI/Fuso-treated group to −15.17 ± 0.88 mV, while the Lipo-treated group produced the most pronounced decrease to −18.82 ± 1.45 mV (Figure 3E). These results suggest that while all formulations modestly influence passive membrane properties in Neuro2A cells, Fuso and PEI/Fuso better preserve membrane resistance and resting membrane potential relative to conventional liposomes. The stronger hyperpolarization observed in the Lipo group may reflect synthetic lipid-induced changes in membrane composition, surface electrostatics, or passive ion permeability.^43–46^ Such hyperpolarization could increase the voltage difference between resting potential and activation threshold, requiring stronger depolarizing input for neural recruitment.

**Figure 3:**
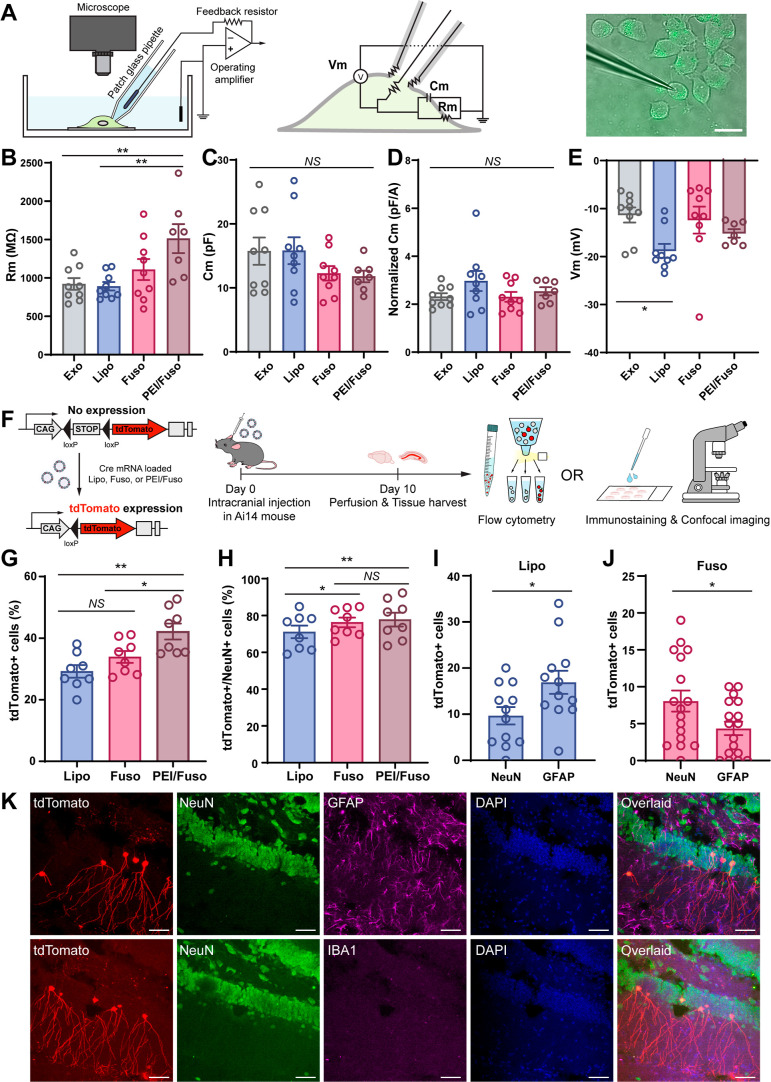
In vitro electrophysiological characterization and in vivo neuron targeting via Fusosomes. (A) Schematic of the patch-clamp technique for in vitro electrophysiological recording (left) and representative brightfield images showing preserved cell membrane morphology across all formulations with patch-clamp electrode attachment (right) (Exo; gray, Lipo; blue, Fuso; pink, PEI/Fuso; magenta). Scale bar: 20 μm. (B–E) Electrophysiological properties of Neuro2A-loxP-GFP cells measured by whole-cell patch clamp recording (*n* = 9 individual cells): (B) membrane resistance (MΩ), (C) membrane capacitance (pF), (D) normalized membrane capacitance per cell surface area (pF/μm^2^), and (E) membrane potential (mV). (F) Experimental schematic showing Cre-mRNA-encapsulated Lipo, Fuso, PEI/Fuso-dependent tdTomato gene expression in Ai14 mice and the timeline for in vivo neuronal targeting analyzed by flow cytometry or by immunostaining and confocal imaging. (G,H) Quantification of in vivo neuronal targeting in Ai14 mice (*n* = 8 whole hippocampi), analyzed by flow cytometry: tdTomato-expressing cell populations across formulations (G) and NeuN-positive cells-expressing tdTomato, identified by anti-NeuN (Alexa Fluor 488) staining (%) (H). (I,J) Quantification of tdTomato-expressing cells from confocal images in Ai 14 mice and their colabeling with NeuN (neurons) or GFAP (astrocytes), confirming the cell types that expressed the delivered Cre mRNA following Lipo (I) or Fuso (J) treatment (*n* = individual confocal images; Lipo (blue), *n* = 12; Fuso (pink), *n* = 17). (K) Representative confocal images showing cellular distribution of tdTomato expression and neuroinflammatory markers following Fusosome delivery in Ai14 mice. tdTomato (red) indicates transgene expression; neurons (NeuN, green), astrocytes (GFAP, magenta, upper), microglia (IBA1, magenta, below), and nuclei (DAPI, blue) are shown. Scale bar, 50 μm. Data presented as mean ± SEM. Statistical significance was determined using one-way ANOVA with Tukey’s multiple comparisons test (B, Exo vs PEI/Fuso **p = 0.0090; Lipo vs PEI/Fuso **p = 0.0057 C, NS *p* = 0.2273 D, NS *p* = 0.2605 E, Exo vs Lipo *p = 0.0373 G, Lipo vs Fuso NS *p* = 0.3116; Lipo vs PEI/Fuso **p = 0.0013; Fuso vs PEI/Fuso *p = 0.0398 H, Lipo vs Fuso *p = 0.0333; Lipo vs PEI/Fuso **p = 0068; Fuso vs PEI/Fuso NS *p* = 0.6957 I, NeuN vs GFAP *p = 0.0311 J, NeuN vs GFAP *p = 0.0373).

To examine whether the Fuso system is sufficient to deliver mRNA cargos in vivo, we performed intracranial injections using stereotaxic coordinates in transgenic Ai14 reporter mice,^47,48^ which express tdTomato fluorescence following Cre-mediated recombination (Figure 3F). Hippocampal tissue from the injection site was collected 10 days after administration of nanocarriers, using the same Cre mRNA dose (0.0125 mg kg^–1^) across all formulations. NeuN staining (neuron-specific antibody) and tdTomato expression in dissociated hippocampal cells were quantified via flow cytometry (Figure S3). The percentages of tdTomato-positive cells were 29.21 ± 2.05% for the Lipo group, 33.90 ± 1.94% for the Fuso group, and 42.14 ± 2.60% for the PEI/Fuso group (Figure 3G).

To assess neuron-specific gene delivery, we quantified the percentage of NeuN-positive cells within the tdTomato-positive population. In the Lipo group, 71.08 ± 3.43% of tdTomato-positive cells were NeuN-positive neurons, which increased to 76.26 ± 2.63% in the Fuso group and 77.76 ± 3.74% in the PEI/Fuso group (Figure 3H). Thus, the Fusosome system supports a higher fraction of neuron-specific targeting, consistent with the distribution patterns observed by confocal fluorescence microscopy (Figure 3I–K).

Immunohistological analysis of hippocampal sections further confirmed these findings. Quantification of tdTomato-expressing cells colabeled with NeuN (neurons) and GFAP (astrocytes) revealed a higher proportion of GFAP^+^/tdTomato^+^ cells in the Lipo group (Figures 3I and S4), whereas in the Fuso group, tdTomato expression was preferentially localized to NeuN^+^ neurons (Figures 3J and S5). High-magnification images further demonstrated the colocalization of tdTomato with NeuN-positive neurons in the Fuso group (Supporting Information Figure S6), supporting enhanced neuronal selectivity in vivo. To further evaluate the local tissue response following intracranial administration, GFAP and IBA1 immunofluorescence staining was performed. Compared with the Lipo group, the Fuso group exhibited lower GFAP and IBA1 fluorescence intensity within the same region of the interest, indicating reduced astrocyte and microglial activation (Figures 3K and S7).

After confirming neuron-specific gene expression, we next tested that mRNA delivery via Fusosome can drive functional neural modulation and produce behavioral changes. We used transgenic Ai32 mice,^49^ which express YFP-tagged channelrhodopsin-2 (ChR2) following Cre recombinase activation and thereby permit optogenetic control of neural activity.^50,51^ Following a similar in vivo Fusosome-induced expression timeline, we intracranially injected 1 μL (mRNA dose 0.0125 mg kg^–1^) of Fuso-Cre into the secondary motor cortex (M2), followed by implantation of an optical fiber at the same site. After 10 days postinjection, the injected mice were subjected to optogenetic stimulation (473 nm, 20 Hz, 10 ms pulse for 3 min) and locomotor behavior testing (Figure 4A). Fluorescence microscopy of M2 tissue around the injection and implantation site confirmed robust ChR2 expression induced by Fuso-Cre delivery (Figure 4B).

**Figure 4:**
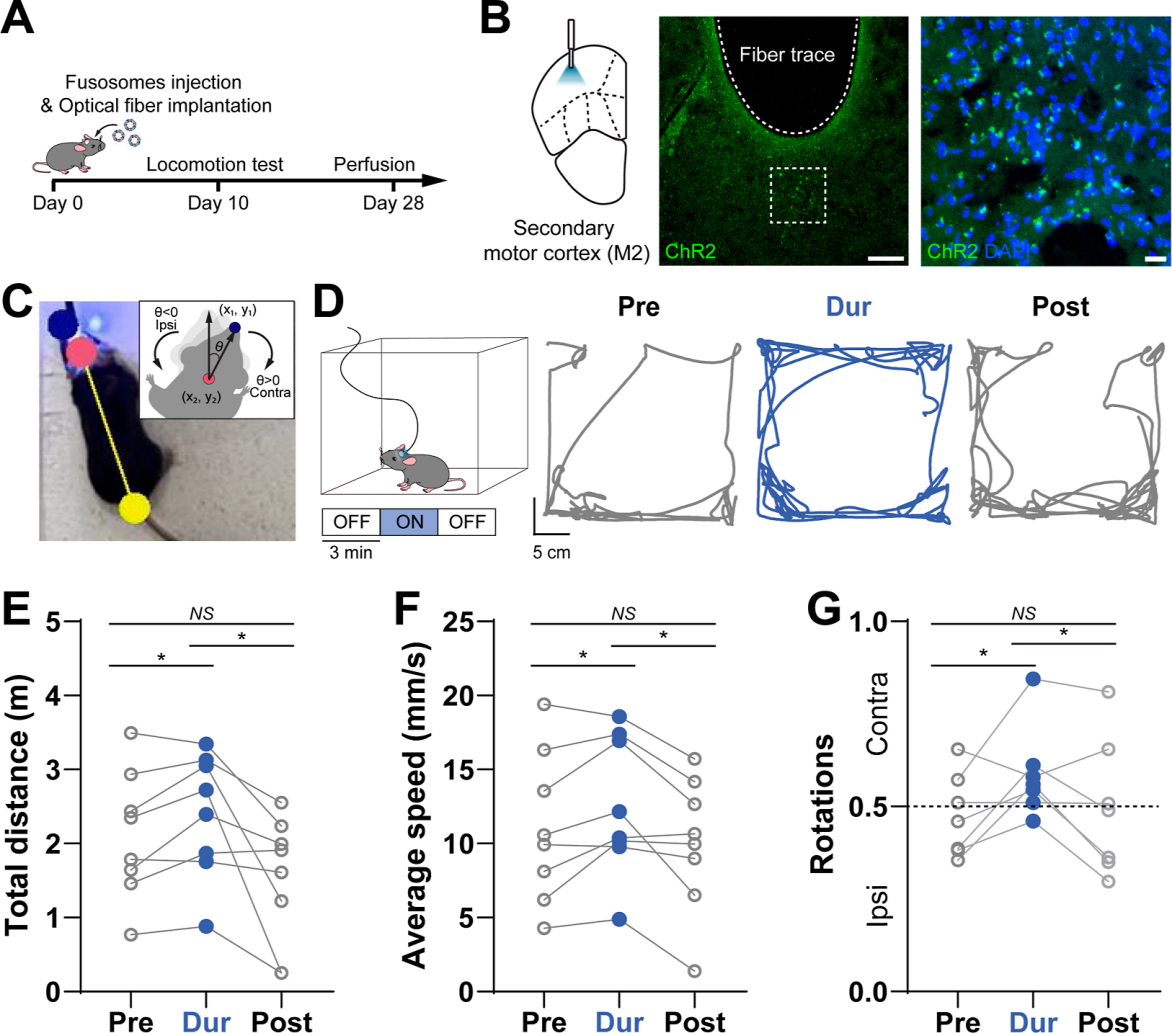
In vivo optogenetic modulation of locomotion in the shallow motor cortex (M2) region via Fusosomes. (A) Schematic of the experimental timeline. (B) Representative confocal microscopy image of YFP-tagged channelrhodopsin-2 (ChR2) expression with optical fiber implantation for light stimulation in Ai32 mice via Fusosome in the M2 region. Scale bar: 50 μm. (C–G) M2 locomotion behavioral analysis (*n* = 8 individual mice, light stimulation protocol: 9 min session with three 3 min epochs (OFF-ON-OFF)): schematic illustration of mouse labeling for head angle calculation and mouse labeling (C), representative mouse trajectories during locomotion (D), total distance traveled (m) (E), average speed (mm/s) (F), and contralateral and ipsilateral rotations (% of total rotations) (G). Statistical significance was determined using a two-tailed paired Student’s *t*-test (E, Pre vs Dur *p = 0.0359; Dur vs Post *p = 0.0227; Pre vs Post *NS p* = 0.081, F, Pre vs Dur *p = 0.0392; Dur vs Post *p = 0.0112; Pre vs Post *NS p* = 0.3378; G, Pre vs Dur *p = 0.0341; Dur vs Post *p = 0.0258; Pre vs Post *NS p* = 0.8742).

Because ChR2 expression was unilaterally induced in M2, we used a markerless video tracking tool (DeepLabCut) to track head, nose, and tail positions over a 9 min locomotor assay (Figure 4C) and to quantify movement trajectories and rotational behavior.^52,53^ We observed increased locomotor activity during optical stimulation (Figure 4D), consistent with quantitative analyses of total distance traveled (Pre: 2.11 ± 0.31 m; Dur: 2.39 ± 0.30 m; Post: 1.50 ± 0.31 m) (Figure 4E) and average speed (Pre: 11.04 ± 1.81 mm/s; Dur: 12.54 ± 1.67 mm/s; Post: 10.01 ± 1.61 mm/s) (Figure 4F). Using an automated algorithm applied to the coordinates extracted from the video tracking (Figures 4C and S8), we further found that mice exhibited a contralateral rotational preference during optical stimulation (Pre: 0.47 ± 0.04; Dur: 0.58 ± 0.04; Post: 0.48 ± 0.06) (Figure 4G). Locomotion analysis showed that Fusosome-driven optogenetic activation produced sustained increases in activity during stimulation, indicating that Fusosome-transduced neurons remain functional and capable of driving behavior.

To test whether Fusosome-mediated gene delivery extends to deep brain circuits that regulate complex behaviors, we next targeted the ventral tegmental area (VTA). Following a similar experimental design, Fuso-Cre (1 μL, mRNA dose 0.0125 mg kg^–1^) were injected into the VTA, followed by implantation of an optical fiber at the same site. Since optogenetic activation of the VTA has been linked to social behavior in mice,^54^ we examined whether Fusosome-induced ChR2 expression in the VTA could modulate social interactions. Ten days after Fusosome injection, mice were subjected to a three-chamber social behavior assay consisting of three phases (prestimulation, stimulation, and poststimulation, 3 min each), with optical stimulation during the middle phase (473 nm, 20 Hz, 10 ms pulses) (social) (Figure 5A). Fusosome-induced ChR2 expression in the VTA was verified by confocal fluorescence microscopy (Figures 5B and Supplementary Figure S9).

**Figure 5:**
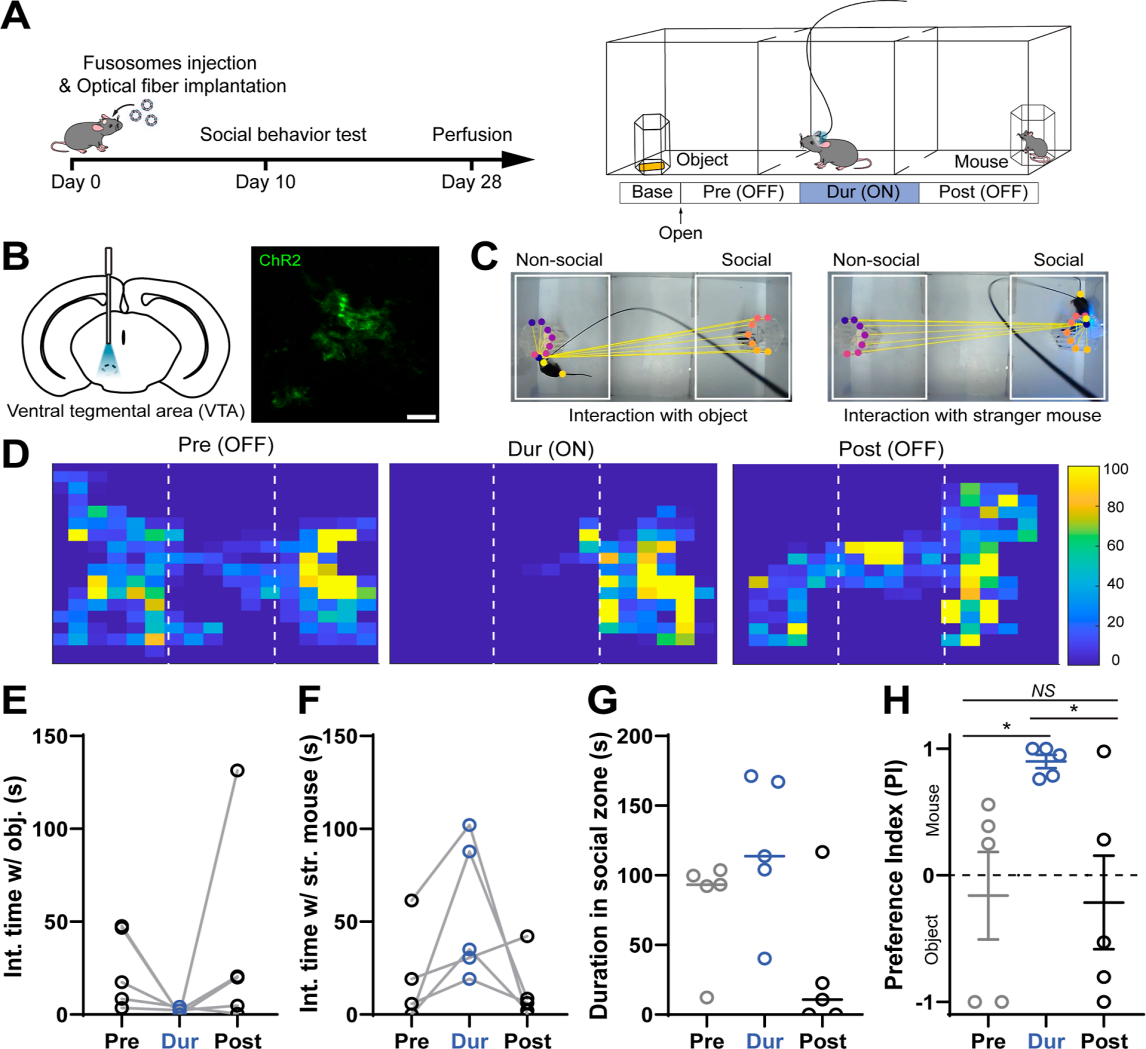
In vivo optogenetic regulation of social interaction in the deep ventral tegmental area (VTA) region via Fusosomes. (A) Schematic of experimental timeline and optical stimulation protocol for social interaction in Ai32 mice: 10 min test divided into four epochs (1 min division and three 3 min epochs with alternating light conditions (OFF-ON-OFF)). (B) Representative confocal image showing ChR2 expression in the VTA region. Scale bar: 50 μm. (C) Representative image showing body part labeling and social zone for DeepLabCut analysis. (D) Representative mouse occupancy heat map during a three-chamber social interaction test. Color scale indicates the number of video frames spent in each spatial bin (0–100 frames at 30 Hz). (E–H) Quantitative assessment of social interaction behavior during optical stimulation, analyzed using DeepLabCut (*n* = 5 individual mice): interaction time (s) with an object (E) and interaction time (s) with a stranger mouse (F), duration in social zone (G), and social preference index (PI) (H). Data presented as mean ± SEM. Statistical significance was determined using a two-tailed paired Student’s *t*-test (Pre vs Dur *p = 0.0473; Dur vs Post *p = 0.0484; Pre vs Post NS, *p* = 0.8264).

To quantify social preference between an object (nonsocial) and a stranger mouse (social), we labeled the experimental mouse and cages and used DeepLabCut to automatically track trajectories and social interactions (Figures 5C and S10). Representative trajectory heatmaps showed an increased preference for the social zone during optical stimulation (Figure 5D). Comparing interaction times with the object and the stranger mouse revealed enhanced social interaction during stimulation. Time spent interacting with the stranger mouse increased from 17.29 ± 11.58 s (Pre) to 54.95 ± 16.69 s (Dur), then decreased to 11.85 ± 7.74 s (Post) (Figure 5F), while object interaction did not show an increase (Figure 5E). A similar pattern was observed when quantifying time spent in the social zone (Pre: 80.28 ± 17.12 s; Dur: 119.3 ± 23.97 s; Post: 30.02 ± 22.09 s) (Figure 5G) and in the social preference index (Figure 5H), indicating that Fusosome-mediated ChR2 expression in the VTA can bias social behavior during optogenetic activation.

## Conclusions

Our results demonstrate that fusosomes preserve essential exosome-derived membrane functions while providing synthetic control over size, charge, and genetic payload, enabling efficient and neuron-preferential gene delivery. The platform supports physiologically intact neuronal responses and drives reliable behavioral effects when interfaced with optogenetic actuators. These features support the utility of fusosomes as a modular tool for interrogating neural circuits. The nanoscale system bridges natural biological specificity with engineering precision, offering controlled gene modulation with a functional output in the nervous system.

## Methods/Experimental Section

### Cell Cultures

The C8-D1A mouse astrocytes (ATCC CRL-2541) and Neuro2A-loxP-GFP cells (a gift from Feng Zhang at MIT) were cultured in Dulbecco’s Modified Eagle’s Medium (DMEM) supplemented with 10% fetal bovine serum (FBS) and 1% penicillin–streptomycin (PS). All cell culture dishes were maintained in a 5.0% CO_2_ incubator at 37 °C.

### Exosomes Isolation

Exosomes were isolated using an ultracentrifugation method. In brief, C8-D1A mouse astrocytes were initially seeded into 150 mm^2^ tissue culture dishes and cultured in DMEM enriched with 10% exosome-depleted fetal bovine serum. The cells were allowed to proliferate for a period of 3 days, promoting the amplification of an abundant cell population and the release of extracellular vesicles. Following this incubation period, the culture medium was harvested and subjected to centrifugation at 5000 rpm for 30 min, effectively removing cellular debris. The supernatant was further refined by passing it through a 0.2 μm vacuum filter to eliminate extra cell debris and large vesicles. To concentrate the extracellular vesicles, the supernatant underwent ultracentrifugation at 33000 rpm for 3 h, and the pellet was washed with PBS and centrifuged at 33000 rpm for 3 h. Subsequently, the pellets were resuspended in PBS solution with 0.001 v/v % Pluronic F-68 (Gibco) and stored at −20 °C for preservation and future use.

### Liposome and Fusosome Preparation

Liposomes were prepared by a standard thin-film hydration and extrusion method. DOTAP (cat. no. 890890), DSPE-PEG2000-NH2 (cat. no. 880128), and DPPC (cat. no. 850355) were purchased from Avanti Polar Lipids. Cholesterol was purchased from Thermo Fisher (cat. no. A11470.30). Lipids (DOTAP/cholesterol/DSPE-PEG2000-NH2/DPPC; molar ratio 2:1.5:0.09:0.5) were dissolved in chloroform. For PEI-containing formulations (PEI/Lipo and PEI/Fuso), chloroform-dissolved branched polyethylenimine (PEI, 25 kDa; Polysciences, cat. no. 23966-1) was added to the lipid solution before thin-film formation at a molar ratio of 0.002 relative to the total lipid content. The solvent was evaporated under reduced pressure, and the resulting lipid (or lipid/PEI) film was dried under a vacuum overnight.

The dried thin film was hydrated with a 10 mM citrate buffer using water-bath sonication. Cre mRNA (50 μL; TriLink Biotechnologies, Cat. No. L-7211) prepared in a 10 mM citrate buffer was immediately added and mixed by vortexing at 1500 rpm for 10 s. For Fusosome formulations (Fuso and PEI/Fuso), purified astrocyte-derived exosomes, quantified by nanoparticle tracking analysis (NTA), were added together with the Cre mRNA during the hydration step at an equal particle number relative to the liposomes (1:1 particle ratio; 5 × 10^10^ particles each) prior to extrusion to enable membrane fusion.

The suspensions were sequentially extruded 20 times through 100 nm (Sterlitech, cat. no. PCT0125100) and 50 nm (Cytiva Whatman, cat. no. 7516574) polycarbonate membranes. The resulting liposomes or Fusosomes were dialyzed against PBS for 2 h using a Pur-A-Lyzer Midi 3500 dialysis tube (Sigma, cat. no. PURD35050-1KT). To remove the citrate buffer following dialysis, the vesicles were centrifuged at 15 000 rpm for 20 min to remove unencapsulated Cre mRNA and resuspended in PBS.

### Liposome and Fusosome Characterization

Transmission electron microscopy (TEM) samples were prepared using negative staining with a drop-to-drop method as follows: 200 mesh carbon-coated copper grids (Electron Microscopy Sciences, Cat no. FCF-200-Cu-50) were placed onto tweezers after the glow discharge step. 10 μL samples were applied to the grid and allowed to incubate for 30–60 s. Afterward, excess liquid was carefully removed from the grid using small sections of filter paper. Subsequently, a UranyLess EM staining solution (Electron Microscopy Sciences, cat no. 22409) was gently applied to the grid and incubated for another 30–60 s. Any excess staining solution was eliminated by blotting with filter paper and further washed three times with Milli-Q water. Finally, the sample deposited grids were air-dried overnight at room temperature. A JEOL JEM-2100F Transmission Microscope was used for TEM sample observation equipped with a field-emission gun 200 kV at different magnifications ranging from 40,000X to 60,000X.

The size and zeta potential of liposomes, fusosomes, and exosomes were measured using a Malvern Zetasizer Nano-ZS, with the samples diluted in DPBS at a 1:50 ratio. The particle concentration of liposomes and exosomes was measured by the Malvern NanoSight NS300 system. To evaluate storage stability, liposomes and fusosome formulations were incubated at 4 °C for different time intervals, and particle size changes were monitored using DLS. Each sample was measured three times for technical accuracy, and the same procedures were repeated. For in vivo functionality testing, the stored fusosome formulation was intracranially injected into Ai14 mice, and tdTomato expression was assessed for 10 days postinjection.

### mRNA Encapsulation Efficiency

mRNA encapsulation efficiency was determined by the Quant-it RiboGreen RNA Assay Kit (Invitrogen, cat no. R11490). Liposomes and Fusosomes were lysed in 0.1% Triton X-100, incubated at RT for 10 min, and diluted to a 1:2000 ratio with TE buffer. Sample fluorescence was measured with a multimode microplate reader (BioTek Synergy Neo 2). The percentage of mRNA encapsulation efficiency (EE %) was calculated using the formula (*F*_total RNA_ – F_measured RNA_/*F*_total RNA_) × 100%. Supplied RNA standards were used to create a standard curve, from which the concentration of encapsulated mRNA was determined. As exosomes inherently contain endogenous RNA, a background correction was performed by preparation of the same amount of exosomes without mRNA loading. The RNA content from these control exosomes was subtracted from the total RNA measured in the mRNA-loaded fusosome formulation to calculate the amount of exogenously encapsulated mRNA.

### In Vitro Transfection Efficacy

Neuro2A-loxP-GFPcells were seeded into 24-well plates containing poly-l-lysine coated coverslips at a density of 5 × 10^4^ cells per well. The old culture medium was then replaced with Opti-MEM and incubated for 2 h prior to transfection. The final mRNA concentration was set at 0.25 μg per well for in vitro transfection. After 48 h of transfection, the cells were fixed with 4 wt % paraformaldehyde (PFA) solution for 30 min on ice. They were then washed three times with PBS and mounted onto glass slides using a DAPI containing mounting medium (Southern Biotech Cat no. 010020) for fluorescence imaging. In vitro transfection was observed using a Nikon Eclipse microscope. GFP-positive cells were counted relative to DAPI-positive cells to calculate in vitro transfection efficiency.

### In Vitro Transfection and Total RNA Preparation for qPCR

Neuro2A-loxP-GFP cells were seeded into 6-well plates at a density of 1 × 10^6^ cells/well. All transfection samples were added to 6-well plates at a 1 μg of RNA per well and incubated for 24 h. Total RNA was isolated from cell lysates using TRIzol (Invitrogen, cat no. 15596026). A MyiQ Real-Time PCR detection system (BIO-RAD) was used for cDNA amplification and SYBR Green Fluorescence detection. Equal amounts of RNA for all samples were used for cDNA amplification (MedChemExpress, Cat no. HY-K0511), and quantitative Real-Time PCR (qRT-PCR) was run on all samples using qPCR master mix (MedChemExpress, Cat no. HY-K0501A) and primers (GAPDH Forward: 5′-ACC ACA GTC CAT GCC ATC AC-3′, Reverse: 5′-TCC ACC ACC CTG TTG CTG TA-3′; GFP Forward: 5′-AGG ACG ACG GCA ACT ACA AG-3′, Reverse:5′-TTC TGC TTG TCG GCC ATG AT-3′) according to the manufacturer’s protocol. Relative mRNA levels were calculated using the 2ΔΔCT method to internally normalize the expression of GFP mRNA to a housekeeping gene, GAPDH.

### In Vitro Biocompatibility

Neuro2A-loxP-GFP were seeded into 24-well plates. 30 μg of lipids of samples (without mRNA) was added to each well. Cell viability was assessed using Calcein-AM (Sigma, Cat no. D5796) to stain live cells, while dead cells were identified with Ethidium Homodimer-1 (Sigma, Cat no. 46043). Imaging was performed using an inverted Nikon Eclipse microscope. Quantitative analysis of dead cell rates was carried out by Fiji software. The dead cell rates were determined as the ratio of dead cell numbers to total cell numbers, calculated using the following formula: Dead cell rates (%) = (number of dead cells/total cell numbers) × 100 (%).

### Whole-Cell Patch Clamp Electrophysiology

Whole-cell patch clamp recordings were conducted on cultured Neuro2A-loxP-GFP cells. Electrophysiological signals were amplified and digitized using a MultiClamp 700B and Digidata1550B (Molecular Devices). A glass patch pipette (Sutter Instrument Borosilicate glass, cat no. BF150-86-7.5) with a resistance of 3 to 8 MΩ was filled with an internal solution (125 mM KCl, 8 mM NaCl, 0.6 mM MgCl_2_, 0.1 mM CaCl_2_, 1 mM EGTA, and 10 mM HEPES, pH adjusted to 7.35 with KOH), with osmolarity adjusted to approximately 295 mOsm using sucrose. The bath solution for the cells consisted of 150 mM NaCl, 5.4 mM KCl, 2 mM CaCl_2_, 2 mM MgCl_2_, 11 mM glucose, and 10 mM HEPES (pH titrated to 7.35 with NaOH) and was adjusted to an osmolarity of 320 mOsm using sucrose.

For voltage-clamp measurements, cells were held at 60 mV. Membrane resistance and cell capacitance were determined by applying 5 mV depolarizing steps in the voltage clamp. Voltage clamp was switched to current clamp, allowing for direct measurement of the membrane potential without input current. The patch-clamped cells are captured after each whole-cell configuration using an Olympus BX61 microscope equipped with a pco.panda 4.2 sCMOS Camera (Excelitas). The cell surface area was measured using ImageJ, and the normalized membrane capacitance was calculated by dividing the capacitance value by the surface area of the cells.

## Animals

All animal experiments in this study received approval from the Institutional Animal Care and Use Committee (IACUC) at the University of Massachusetts Amherst (Protocol no. 2476) and Binghamton University—The State University of New York (protocol no. 895-23) and were conducted in strict adherence to relevant regulations and guidelines. Ai14 mice (B6.Cg-Gt­(ROSA)­26Sortm14­(CAG-tdTomato)­Hze/J, JAX stock no. 007914) and Ai32 (B6.Cg-Gt­(ROSA)­26Sortm32­(CAG-COP4*H134R/EYFP)­Hze/J, JAX stock no. 024109) were supplied from The Jackson Laboratory (JAX). These mice, aged 6–8 weeks old and weighing 20–25 g, were housed in cages with no more than 5 mice per cage, providing them with free access to food and water.

### Intracranial Stereotaxic Injection

All mice were anesthetized using a chamber with 1.5% isoflurane, then placed onto a stereotactic frame with an isothermal pad. All surgical procedures were performed in sterile conditions with 1.0% isoflurane to maintain anesthesia. A micro drill (RWD) made a small hole in the skull at the injection location. The Allen Brain Atlas was utilized to determine the coordinates for the injection site, targeting the hippocampal cornu ammonia (CA1) AP: −2.0 mm, ML: ±1.5 mm, DV: −1.5 mm; the secondary motor cortex (M2) AP: 1.5 mm, ML: ±0.5 mm, DV: −1.5 mm; ventral tegmental area (VTA) AP: −2.95 mm, ML ± 0.5 mm, DV: −4.5 mm. Fusosomes were injected into CA1 for cell-type specificity and into M2 and VTA for animal behavioral analysis. An injection volume of 1000 nL of liposomes and fusosomes was delivered to the specified coordinates using a microinjection syringe pump (World Precision Instruments, MICRO 2T SMARTouch controller) equipped with NANOLITER2020 and a glass-pulled pipet at a controlled rate of 200 nL per minute. The syringe was left in place for 5 min at each injection site to enhance diffusion and prevent the inadvertent withdrawal of injected samples. The incision area was closed using a 5.0 nylon suture (Ethicon 661H). The final RNA amount administered was 250 ng per 20 g mouse (up to 2 μL), corresponding to a dose of 0.0125 mg/kg body weight.

### Flow Cytometry for Cell Specificity

Mice were euthanized via intraperitoneal injection of pentobarbital sodium and phenytoin sodium (200 mg/kg). They were then transcardially perfused with PBS to remove blood. The brain tissues were carefully dissected in cold PBS, and the hippocampus was isolated. The tissue was finely minced on a flat Petri dish containing cold 0.1 v/v % Tween20 in PBS (PBST20) using sharp razor blades. The minced tissues were transferred into a microfuge tube with PBST20 with a 1 mL syringe, and the suspension was passed through a 19G needle back and forth 12 times. The suspension was then centrifuged at 250*g* for 3 min, and the supernatant was discarded. To digest the tissue, 1 mL of enzyme solution (collagenase and trypsin) was added to the tissue pellet, and the mixture was shaken at 37 °C for 1 h at 200 rpm. After digestion, the tissue was centrifuged again at 250*g* for 3 min, and the supernatant was discarded. The pellet was washed once more with cold PBST20 to remove excess enzymes. Subsequently, the tissue pellet was titurated four times in cold PBST20, starting with 12 times passing through a 21G needle. The mixture was placed on ice for 5 min, and the cloudy supernatant was removed. An additional PBST20 was added to the remaining tissue for another trituration with a 23 G needle and then 25G needle. All supernatants were combined and filtered through a 70 μm mesh. The cell suspension was fixed in 4% paraformaldehyde (PFA) in PBS for 30 min on ice, then centrifuged and resuspended in PBS for flow cytometry analysis.

To quantify the number of neurons, an anti-NeuN antibody (NeuN Monoclonal Antibody (3A4C1), CoraLite Plus 488 Thermo Scientific, cat no. CL488-66836) was added to the cell suspension at a 1:200 ratio and incubated in the dark for 30 min. The stained cell suspension was then centrifuged at 250g for 5 min and resuspended in PBS containing 0.1 wt % BSA. All samples were analyzed using the Cytek Aurora CS system.

### In Vivo Optical Fiber and Optrodes Implantation

Optical fiber and optrodes positioned at the designated coordinates for optogenetic stimulation in Ai32 mice after intracranial injection (*n* = 8, M2 for locomotion; *n* = 5, VTA for social interaction). The optical fiber was prepared using a Ø400 μm core multimode silica fiber (Thorlabs, cat no. FP400URT) and a 440 μm ceramic optical ferrule. The devices were attached to the skull using dental adhesives (Parkell C&B Metabond and Lang Dental Manufacturing Company, Inc. Jet Set-4).

### Mice Behavioral Analysis with Optogenetic Stimulation

Mice were habituated in the behavioral chamber for 30 min prior to recording, 7 days after injection and implantation. Optical fibers were connected to a blue light 473 nm laser. Optical stimulation was controlled by a function generator set to 26 mW mm^–2^, 20 ms pulses at 20 Hz. The optical stimulation protocol in M2 regions consisted of 3 min of light off (prestimulation), 3 min of light on (stimulation), and 3 min of light off (poststimulation) for locomotion assessment in total 9 min video recording. The recorded videos were analyzed using DeepLabCut (DLC) software to track the precise coordinates of the mice by identifying anatomical landmarks on the head, nose, and tail during each epoch. This enabled automatic tracking of the mouse’s location. The DLC analysis provided detailed location data, which was further processed using a custom-written MATLAB algorithm. From the DLC-tracked coordinates, the following behavioral metrics were extracted: total traveled distances, average speed, mice trajectories, and head angles.

For social interaction, mice underwent a 30 min open field habituation, which is the same size of three chambers, 30 min habituation period, followed by a 10 min pretest, and then a 10 min recording session. During the social interaction test, mice were separated by a divider in a three-chambered box to isolate them from unfamiliar mice and objects for 1 min. The total recording time included 9 min of pre-, during, and poststimulation. The recorded videos were analyzed using DLC software (version 2.2) to identify anatomical landmarks, including mouse’s nose, tail, implanted device, and the cage containing an unfamiliar mouse. A custom-written MATLAB algorithm sorted the mouse’s interaction time with the object and the unfamiliar mouse, as well as to generate heat maps of time spent in the chamber, based on DLC tracking coordinates.

### Immunostaining

For the cross sectioning, the brains were incubated in a 30 wt % sucrose solution at room temperature for 2 days and then were embedded in the OCT compound (Fisher Scientific Tissue-Plus cat no. 23-730-571) and sectioned into 20 μm thick slices using a cryostat.

The antibodies were diluted in a blocking solution comprising 0.5 v/v % Triton-X and 1 wt % bovine serum albumin in PBS. Brain tissue slides were placed in a 60 °C heating oven for 2 h to prevent tissue detachment during the immunostaining procedure. Following this heat treatment, the tissues were cooled down at room temperature and rinsed three times with a PBS solution containing 0.5 v/v % Triton-X (PBST) and then incubated in a blocking solution for 30 min. Subsequently, they were incubated with the primary antibody for 2 h, followed by a 1 h incubation with the secondary antibody. The slides underwent two washes with PBST solution and one wash with Milli-Q water before being mounted with a solution containing DAPI.

The stained slides were then imaged using a confocal laser scanning microscope, specifically the Zeiss LSM 880 model, which is equipped with four laser lines, six excitation wavelengths, three PMT detectors, and a 20X objective lens. Image stacking and quantitative analysis were performed using Fiji software.

### Statistical Analysis

All statistical analyses were performed using GraphPad Prism 10. For multiple comparisons, one-way ANOVA was applied, and if the resulting *p* < 0.05, a Tukey’s multiple comparisons test was conducted to compare each pair of groups. For animal behavioral analysis, a two-tailed paired Student’s *t*-test was performed to calculate p-values. All experiments were carried out in at least three independent trials, unless otherwise noted.

## Supplementary Material







## Notes

Video 1: Use of a markerless video tracking tool (DeepLabCut) to track head, nose, and tail positions over a 9 min locomotor assay and to quantify movement trajectories and rotational behavior (MP4)
Video 2: To quantify social preference between an object (nonsocial) and a stranger mouse (social), labeling of the experimental mouse and cages and use of DeepLabCut to automatically track trajectories and social interactions (MP4)
Exosome-mediated GFP mRNA expression; time-dependent Cre mRNA delivery; flow cytometry gating; neuronal specificity of tdTomato expression; local astrocytic and microglial responses; DeepLabCut- and MATLAB-based locomotion, rotation, and social-interaction analysis workflows; and ChR2-EYFP expression in the ventral tegmental area (Figures S1–S10) (PDF)
