# Supplementary material for: Hybrid Nonviral Nanocarriers Enable Functional Neural Modulation
Source: ACS Nano. 2026 Jul 22;20(30):21376–87. doi: 10.1021/acsnano.6c07588 (PMC13450448; doi:10.1021/acsnano.6c07588)
Supplement: Supplementary file 1 [file nn6c07588_si_003.pdf]

Supplementary Materials for

Hybrid Non-viral Nanocarriers Enable Functional Neural Modulation

Eunji Hong[1], Xinxin Xu[1], Sizhe Huang[1], Geunho Jang[1], Zuer Wu[1], Qianbin Wang[1,*], Siyuan Rao[1,2,*]

Corresponding author: Qianbin Wang (qbwang@binghamton.edu) and Siyuan Rao (syrao@binghamton.edu)

The Supporting Information includes:

Figs. S1 to S10

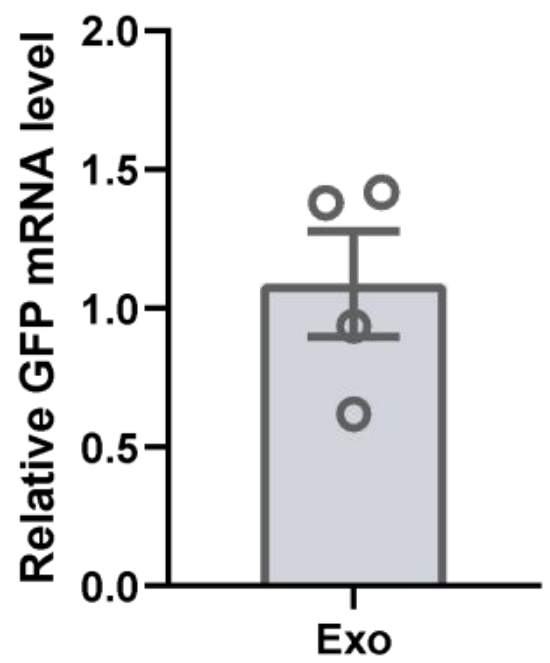

Fig. S1. Relative GFP mRNA levels in Neuro2A-loxP-GFP cells treated with astrocyte-derived exosomes (Exo). Ct values were obtained for GFP and the housekeeping gene GAPDH, and mean Ct values were calculated from technical triplicates. ΔCt was calculated as $\Delta Ct = Ct_{GFP} - Ct_{GAPDH}$. ΔΔCt was calculated relative to untreated control cells: $\Delta\Delta Ct = \Delta Ct_{sample} - \Delta Ct_{control}$. Fold changes in GFP mRNA expression were determined using Fold change = $2^{-\Delta\Delta Ct}$. All values were normalized to untreated control cells (set to 1). Data are presented as mean ± S.E.M. (n = 4 individual samples).

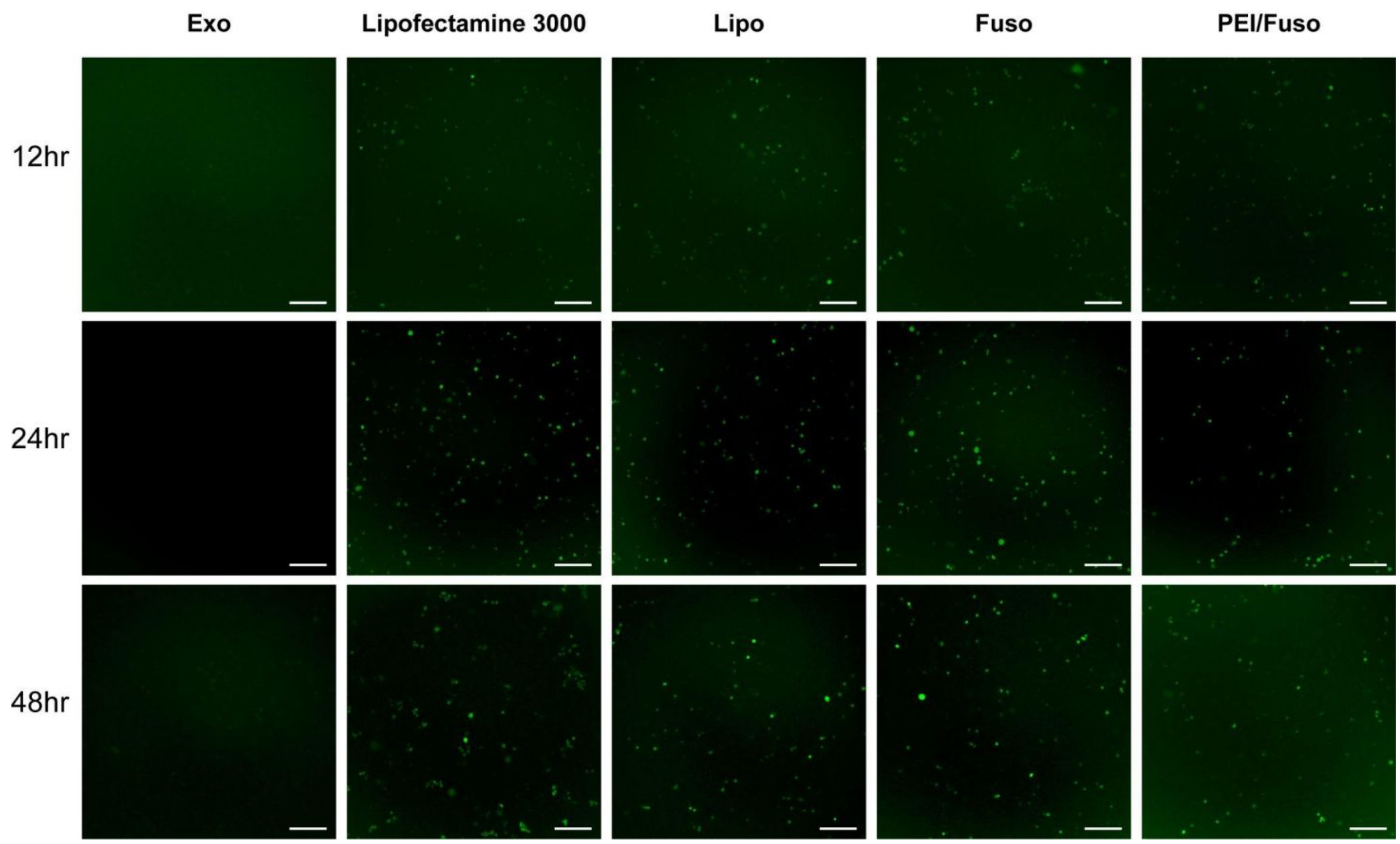

Fig. S2. Representative fluorescence images showing time-dependent Cre mRNA-induced GFP expression in Neuro2A-loxP-GFP cells at 12, 24, and 48 h after transfection. Cells were treated with Exo, Lipofectamine 3000, Lipo, Fuso, or PEI/Fuso loaded with Cre mRNA (0.25 µg Cre mRNA per well in a 24-well plate). Scale bar, 100 µm.

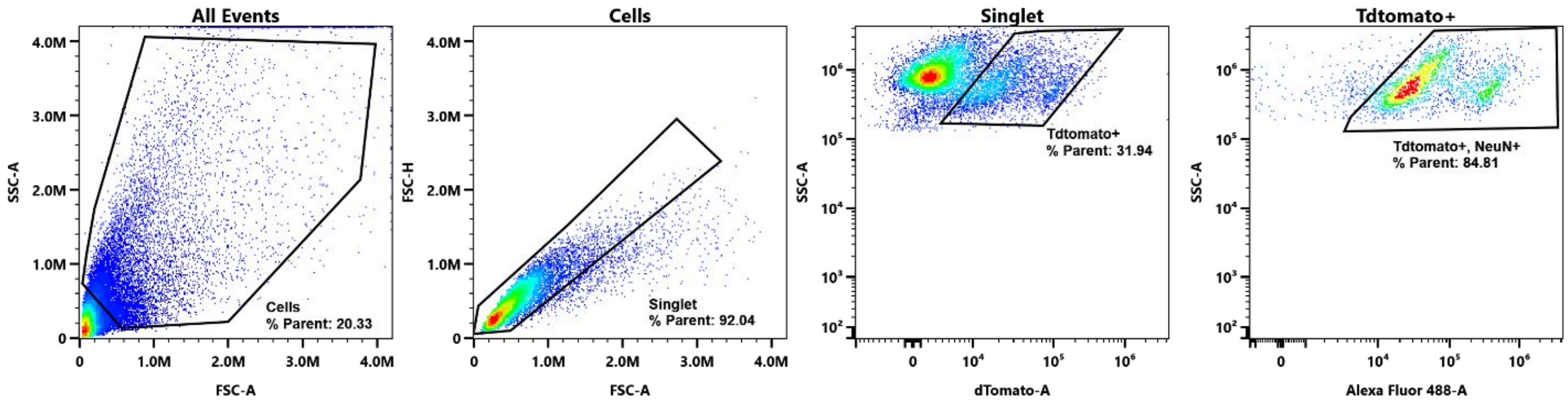

Fig. S3. Flow cytometry gating strategy for isolating NeuN-positive and tdTomato-positive cell populations. A total of 50,000 events were gated on FSC and SSC, with 20% (approximately 10,000) to exclude debris and dead cells then singlets were gated on FSC. Singlets retained to quantify tdTomato-positive cells and NeuN-labeled tdTomato-positive cells. Finally, tdTomato- and NeuN-positive cells were gated based on untreated controls.

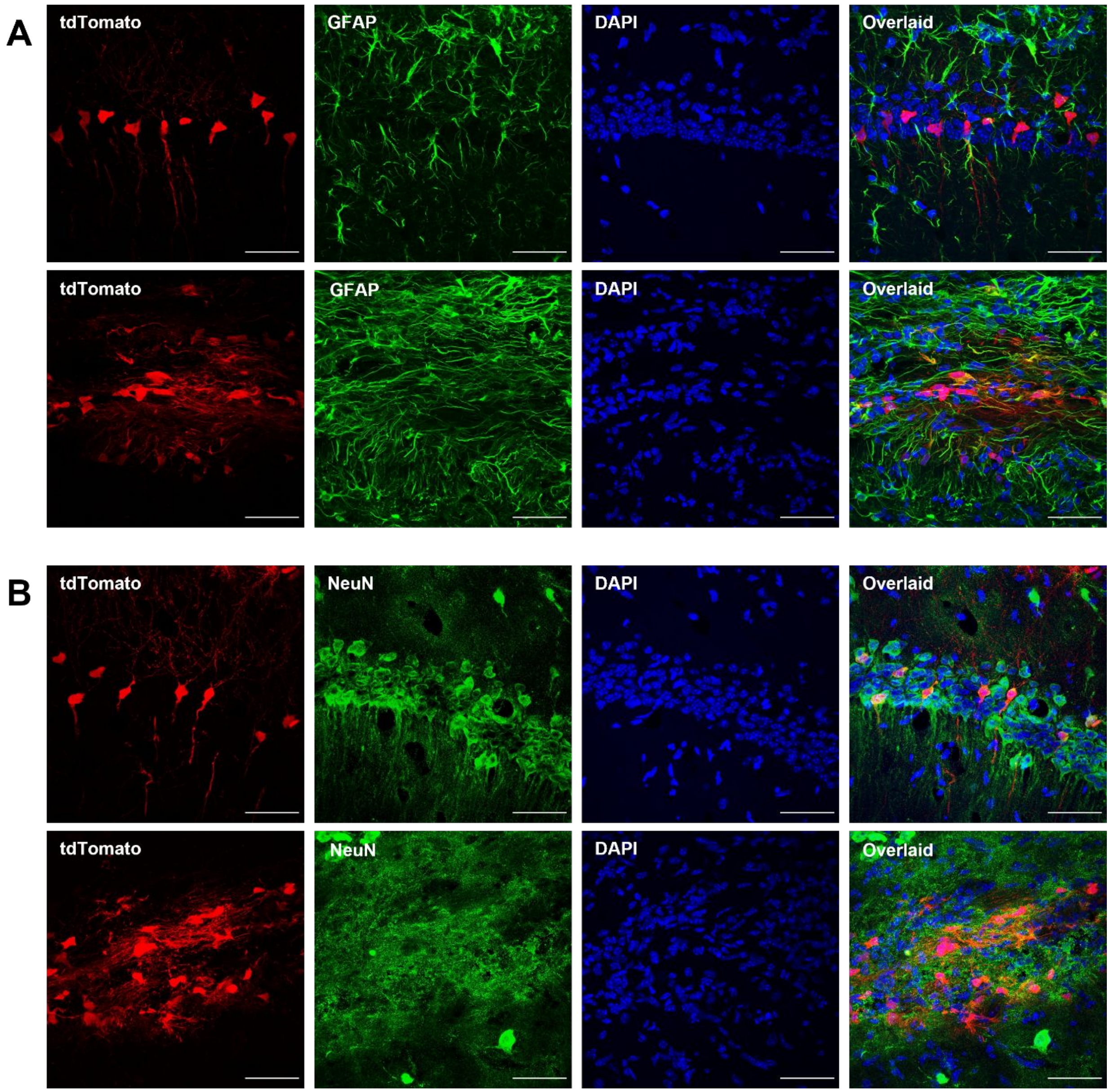

Fig. S4. Representative confocal images of neuron-specific tdTomato expression via Lipo treatment in Ai14 mice. tdTomato (red) expression shown with immunostaining for neurons (NeuN, green) in (**A**) and astrocytes (GFAP, green) in (**B**); nuclei are counterstained with DAPI (blue). Scale bar: 50 µm.

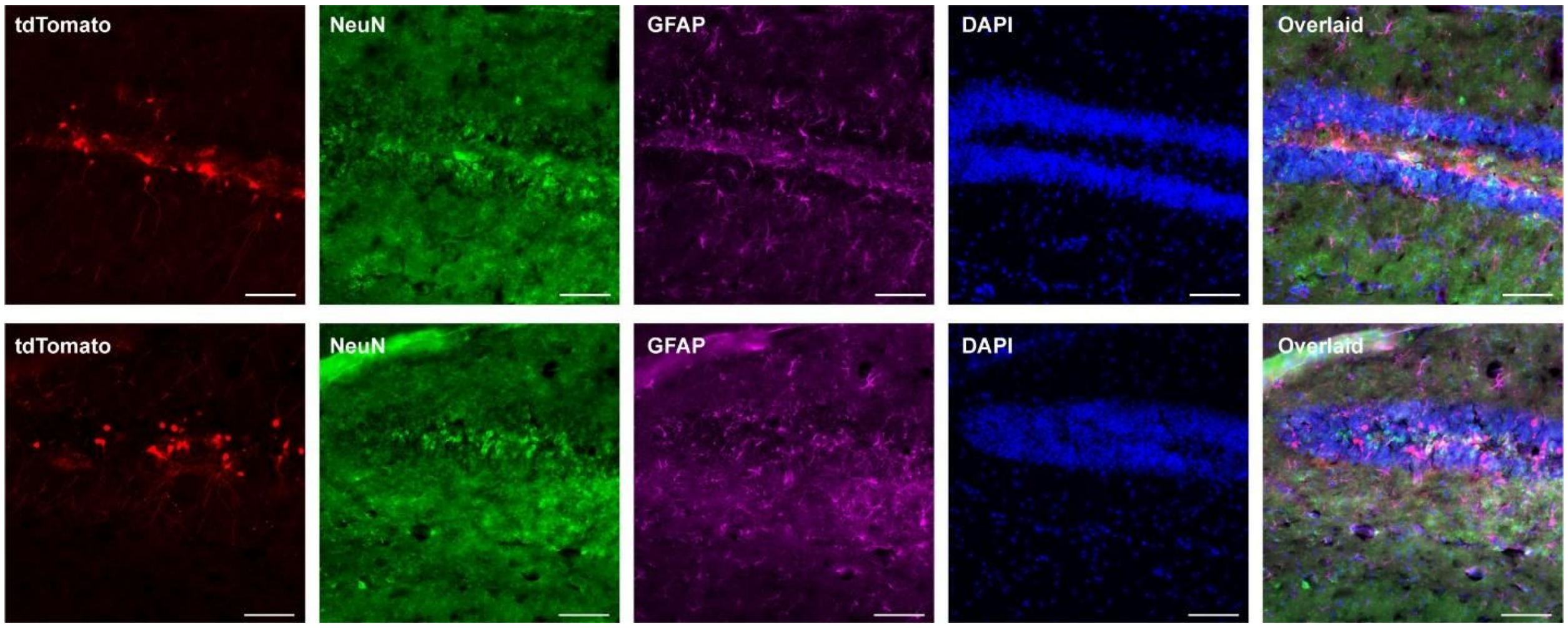

Fig. S5. Representative confocal images of neuron-specific tdTomato expression via Fuso treatment in Ai14 mice. tdTomato (red) expression shown with immunostaining for neurons (NeuN, green), astrocytes (GFAP, magenta), and nuclei (DAPI, blue). Scale bar: 100 µm.

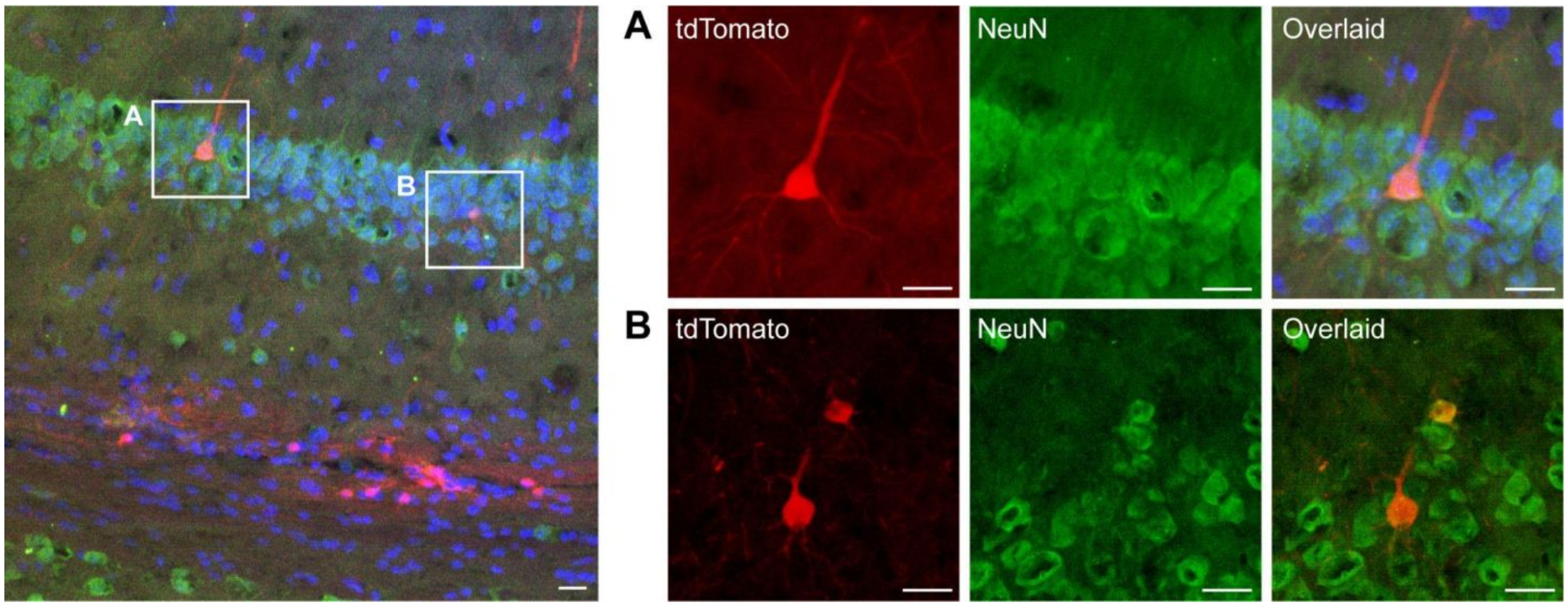

Fig. S6. Representative confocal image demonstrating tdTomato expression in Ai14 mice after Fuso injection. The left panel shows a low-magnification overview image with regions of interest (**A** and **B**) indicated by white boxes. Enlarged views of regions **A** (upper right) and **B** (lower right) reveal the detailed morphology of tdTomato-positive cells (red) and their co-localization with NeuN (green). Scale bar, 20 μm.

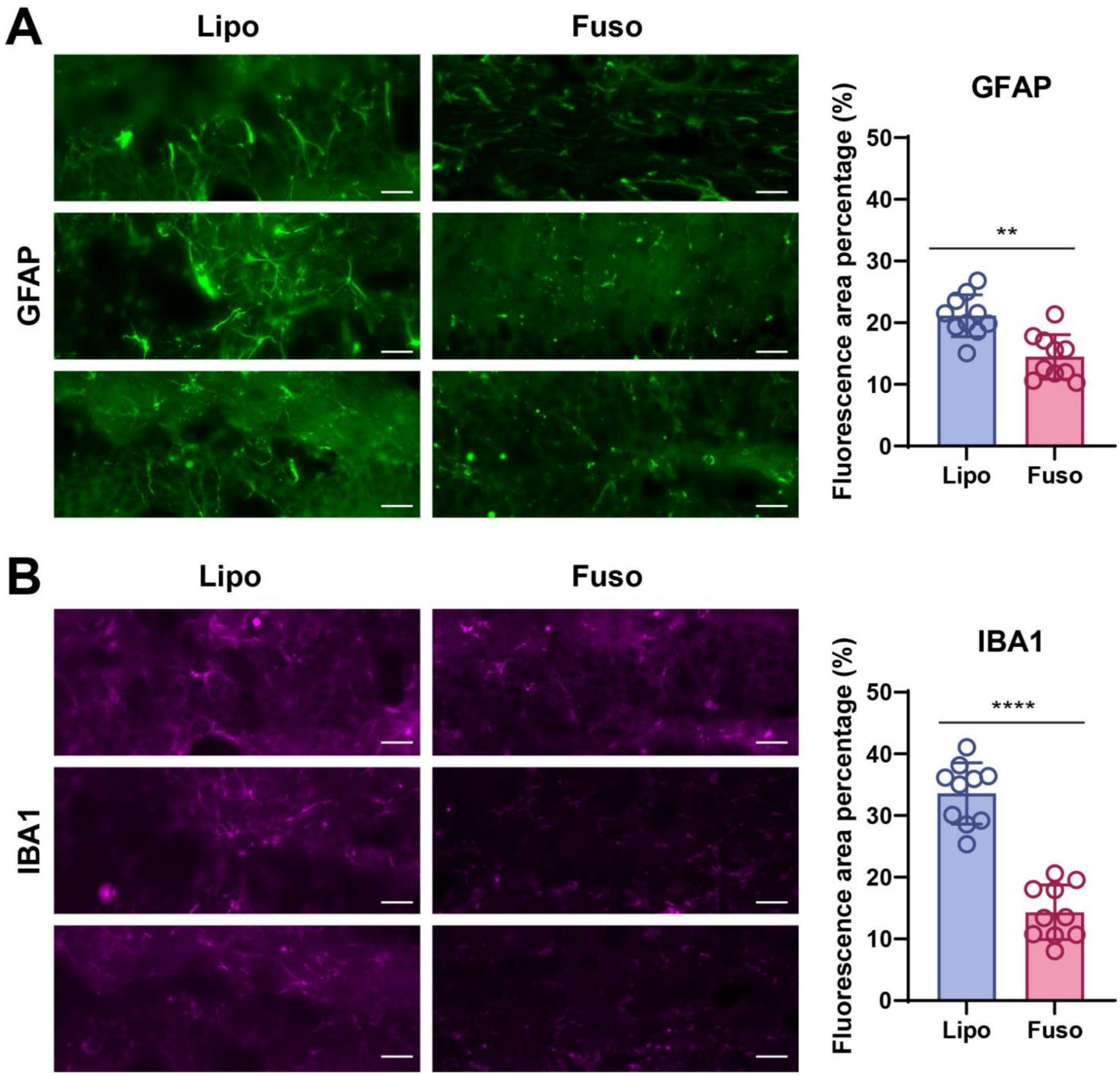

Figure S7. Comparison of the local immune response following Liposome and Fusosome injection. (A) Representative fluorescence images of astrocytes labeled with GFAP (green) surrounding the injection sites following Liposome or Fusosome administration (left) and corresponding quantification of the GFAP-positive fluorescence area (right). **(B)** Representative fluorescence images of microglia/macrophages labeled with IBA1 (magenta) surrounding the injection sites (left) and corresponding quantification of the Iba1-positive fluorescence area (right). Fluorescence area was quantified from regions of interest (ROIs; 266.25 × 266.25 μm) adjacent to the injection sites. Data are presented as mean ± standard deviation (SD). Each hollow dot represents the average fluorescence area measured from one ROI image (n = 10 ROI images from 3 mice per group). Statistical significance was determined using a two-tailed Mann-Whitney test. GFAP: P = 0.0011; Iba1: **P < 0.0001. Scale bar, 50 μm.

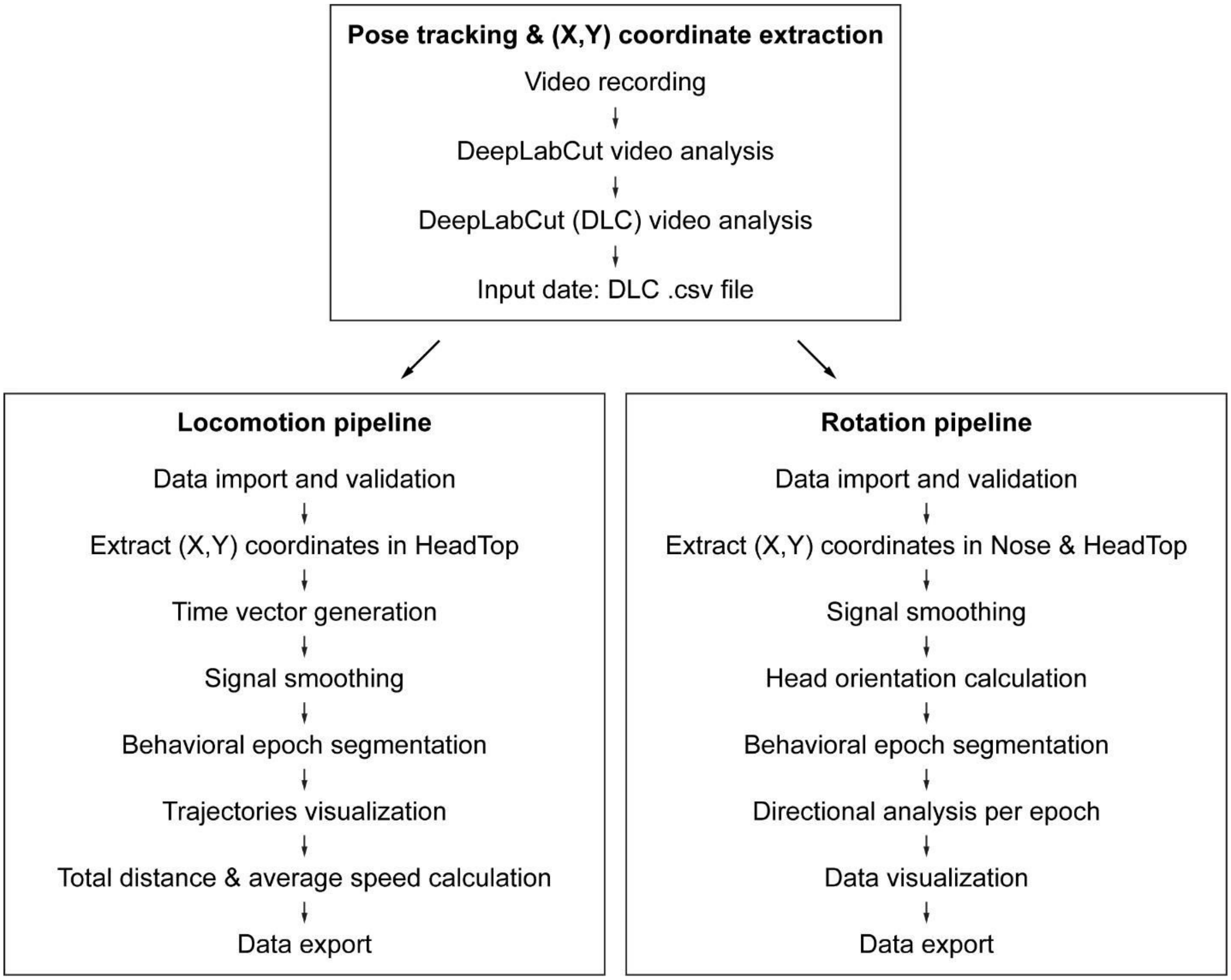

Fig. S8. Workflow diagram summarizing the DeepLabCut- and MATLAB-based locomotion behavioral test video analysis pipeline. DeepLabCut tracking provides Nose, Head Top, and Tail XY coordinates, which are imported into MATLAB, smoothed, and segmented into pre-, during-, and post-stimulation epochs. The workflow then separates into two branches. **Left, locomotion analysis.** Smoothed HeadTop coordinates are used to compute mouse trajectories, total travel distance (sum of frame-to-frame displacement), and average speed (distance divided by epoch duration). These values are quantified separately for the pre-, during-, and post-stimulation periods. **Right, rotation analysis.** Nose-HeadTop vectors are used to calculate instantaneous head angles at each frame (atan2d of the y- and x-axis differences). Angular time series are then used to classify ipsilateral vs contralateral rotations based on the sign of angular velocity.

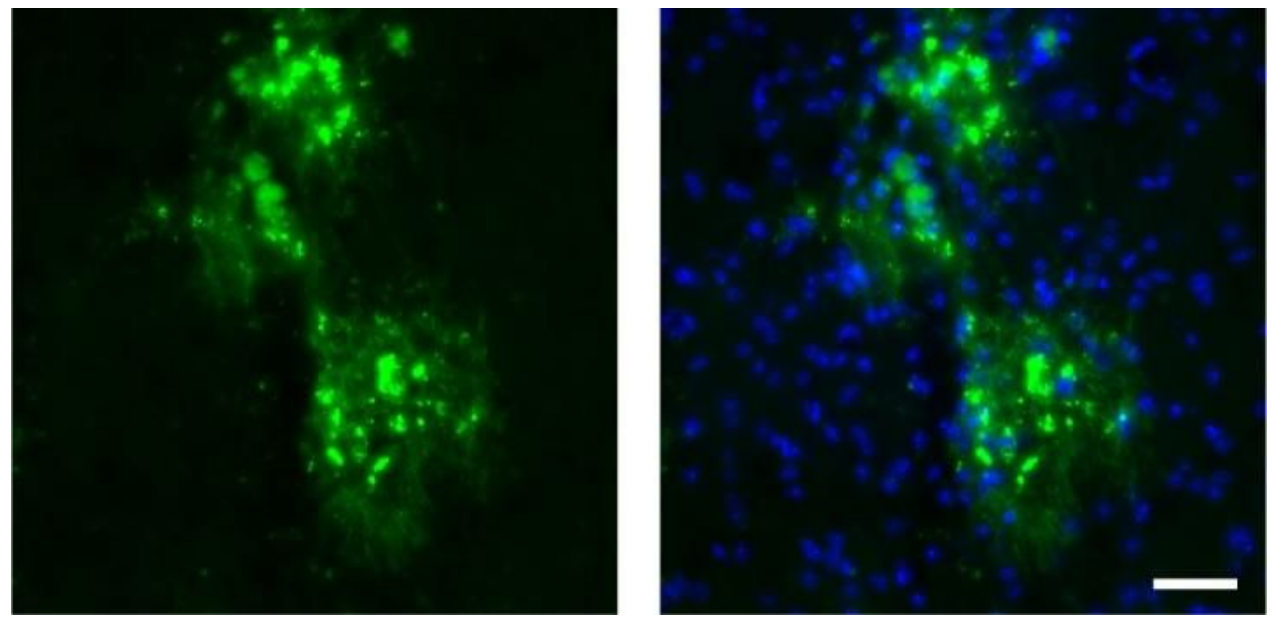

Fig. S9. Representative fluorescence images of ChR2-EYFP expression in the ventral tegmental area (VTA) of Ai32 mice (left, ChR2-EYFP; right, overlay with DAPI). Scale bar: 50 μm.

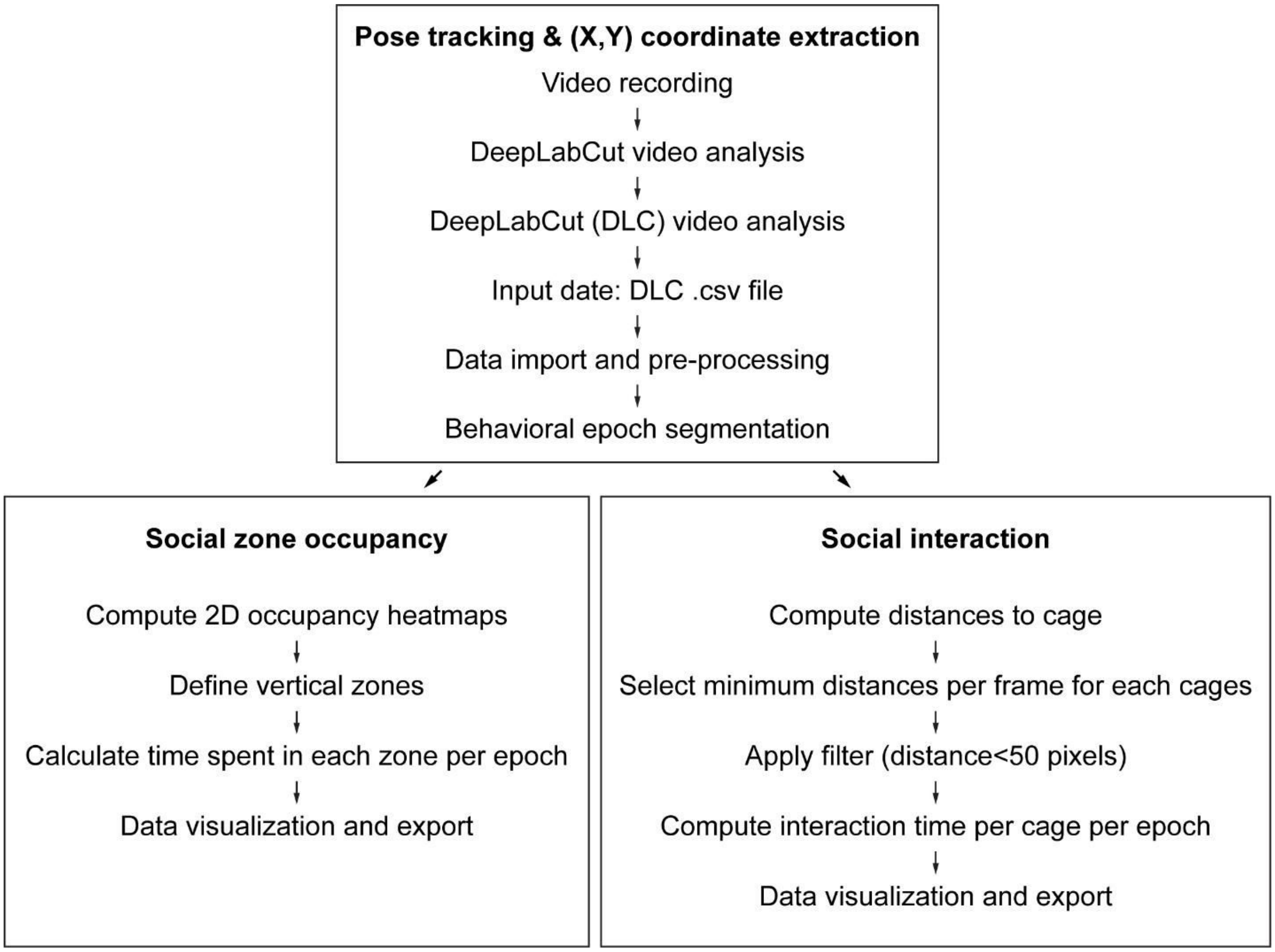

Fig. S10. Workflow diagram summarizing the DeepLabCut- and MATLAB-based social interaction behavioral test video analysis pipeline. Raw videos first proceed with DeepLabCut to extract XY coordinates and likelihoods for the Nose, HeadTop, Tail, and cage markers. The data preprocessed by filtering coordinates with low likelihood (<0.1), restricting positions to arena boundaries (X: 150-1100, y:100-600), and interpolating missing values. The time series are then segmented into four epochs: Baseline, Pre-, During-, Post-stimulation. The pipeline splits into two parallel branches. **Left, Heatmap and social zone analysis.** Nose trajectories are binned to generate 2D occupancy heatmaps, and the arena is divided into three equal-width vertical zones to quantify time spent in non-social, center, and social zones. **Right, Social interaction analysis.** Frame-by-frame distances between the mouse (Nose and Tail) and cage markers select minimum distances for the object and stranger mouse cages, apply filters (Nose closer than Tail, minimum likelihood, distance <50 pixels), and calculate interaction time per cage for each epoch, with optional interaction timelines. The final outputs include heatmaps, zone-occupancy measures, and time-resolved social interaction quantification.
